# Effect of Damping and Yielding on the Seismic Response of 3D Steel Buildings with PMRF

**DOI:** 10.1155/2014/915494

**Published:** 2014-07-06

**Authors:** Alfredo Reyes-Salazar, Achintya Haldar, Ramon Eduardo Rodelo-López, Eden Bojórquez

**Affiliations:** ^1^Facultad de Ingeniería, Universidad Autónoma de Sinaloa, Ciudad Universitaria, 80040 Culiacán, SIN, Mexico; ^2^Department of Civil Engineering and Engineering Mechanics, University of Arizona, Tucson, AZ 85721, USA

## Abstract

The effect of viscous damping and yielding, on the reduction of the seismic responses of steel buildings modeled as three-dimensional (3D) complex multidegree of freedom (MDOF) systems, is studied. The reduction produced by damping may be larger or smaller than that of yielding. This reduction can significantly vary from one structural idealization to another and is smaller for global than for local response parameters, which in turn depends on the particular local response parameter. The uncertainty in the estimation is significantly larger for local response parameter and decreases as damping increases. The results show the limitations of the commonly used static equivalent lateral force procedure where local and global response parameters are reduced in the same proportion. It is concluded that estimating the effect of damping and yielding on the seismic response of steel buildings by using simplified models may be a very crude approximation. Moreover, the effect of yielding should be explicitly calculated by using complex 3D MDOF models instead of estimating it in terms of equivalent viscous damping. The findings of this paper are for the particular models used in the study. Much more research is needed to reach more general conclusions.

## 1. Introduction

Because of our limited knowledge about the* Earthquake Phenomenon*, seismic analysis and design procedures for structures are updated or modified on a continuous basis. Several methods with different degrees of sophistication have been suggested in most codes. They include the static equivalent lateral force (SELF) procedure, the nonlinear static procedure (PUSHOVER), and several types of dynamic analysis procedures like modal response, spectral, linear time-history, and nonlinear time-history analyses. Even though in current building codes the inelastic behavior of structures is explicitly considered by using nonlinear methods, shifting away from the traditional elastic analysis, the use of simplified methods like SELF procedure is still broadly used. Many seismic building codes around the world permit the use of this procedure for regular structures with relative short periods.

According to the SELF procedure, buildings are designed to resist seismic equivalent static lateral forces which are related to the seismicity of the region and the type of structure under consideration. Some equations are given to estimate the base shear and the distribution of lateral forces over the height of the building. Static analysis of the building acting upon these forces provides the design forces. In the procedure, the elastic base shear is reduced by using a factor, here called seismic reduction factor (*R*) which mainly depends on the structural overstrength and the energy dissipation capacity which in turn depends on the structural system, structural material, and level of detailing. As a consequence of the procedure, the reduction in resultant stresses, say axial load or bending moments in columns, is reduced in the same proportion as that of base shear. The reduction is supposed to be particularly important for steel structures since energy dissipation is supposed to come from different sources.

Many mechanisms contribute to the energy dissipation in actual building structures. As it will be additionally discussed, they have an important effect on the structural responses. In the case of seismic analysis of steel buildings, this dissipation is usually considered in two ways: an equivalent viscous damper is used to model the energy dissipation at deformations within the elastic limit of the structure while the dissipated energy due to inelastic behavior (yielding) of the material is considered by including the inelastic relationship between resisting forces and deformation. As it will be discussed in more detail in the subsequent section, the effect of the energy dissipated by each of these mechanisms on the structural response has been studied for simplified structural systems but not for complex structural representations. The evaluation of the effect of damping and yielding on the seismic response of 3D steel buildings with perimeter moment resisting frames, modeled as complex multidegree of freedom (MDOF) systems, constitutes the primary objective of this study. The effect is expressed in terms of the reduction of global and local response parameters.

## 2. Literature Review

There have been many investigations regarding the estimation of the dissipated energy as well as its effect on the seismic response of steel buildings and the related force reduction and ductility factors. One of the first investigations was conducted by Newmark and Hall [1]. They proposed an approximated procedure for constructing the inelastic response spectra from the basic elastic design spectra by relating the seismic reduction and the ductility parameters. Hadjian [2] studied the reduction of the spectral accelerations to account for the inelastic behavior of structures. Nassar and Krawinkler [3] studied the relationship between force reduction factors and ductility for SDOF and simplified (three-story single-bay) MDOF systems. Miranda and Bertero [4] proposed simplified expressions to estimate the inelastic design spectra as a function of the maximum tolerable ductility, the period of the system, and the soil conditions of the site. Shen and Akbas [5] proposed simplified expressions to estimate the input energy and the damping energy of steel moment resisting frames subjected to a group of ground motions recorded on different types of soils. Santa-Ana and Miranda [6] studied the strength reductions factors for several steel frames modeled as plane MDOF systems considering different soil conditions. Borzi and Elnashai [7] derived values of the strength reduction factors needed for predetermined levels of ductility. Reyes-Salazar and Haldar [8] by using simplified plane models found that the dissipation of energy produced by viscous damping, or by yielding of the material, is comparable to that of partially restrained connections. Reyes-Salazar [9] studied the ductility capacity of plane steel moment resisting frames; local, story, and global ductility were considered. It was shown that using SDOF systems to estimate the ductility capacity may be a very crude approximation. Ramirez et al. [10] presented the derivation of the 2000 NEHRP simplified methods for calculating the maximum acceleration and maximum velocity in damped framing systems. In another investigation, Ramirez et al. [11] studied the effect of damping on the response of elastic and inelastic SDOF systems by using earthquake histories that matched on average a 2000 NEHRP spectrum on a stiff soil site for a region of high seismicity. Ramirez et al. [12] proposed an equivalent lateral force and modal analysis procedures for yielding buildings with damping systems which were incorporated in the 2000* NEHRP Provisions*. Arroyo-Espinoza and Teran-Gilmore [13] from the study of the dynamic response of SDOF systems proposed expressions to estimate strength reduction factors that should be used to reduce the elastic response spectra to establish the design seismic forces for structures with different combinations of plastic and viscous energy dissipating capacities. Hong and Jian [14] studied the impact of the uncertainty in the natural vibration period and the energy dissipated by damping on the peak displacement of linear elastic and elastoplastic SDOF systems. Zhai and Xie [15] proposed an expression to estimate strength reduction factors considering several parameters concluding that the effects of site conditions and classification of design earthquakes cannot be neglected while the magnitude and distance of the earthquakes do not have practical effects. Levy et al. [16] used an equivalent linearization approach to derive approximate harmonic equivalent stiffness and damping for bilinear systems in the context of earthquake resistant design and used the results to evaluate the strength reduction factor for given ductility. Karmakar and Gupta [17] performed a parametric study to estimate the dependence of strength reduction factors on strong motion duration, earthquake magnitude, geological site conditions, and epicentral distance for elastoplastic oscillators. Cai et al. [18] estimated the ductility reduction factors for MDOF systems by modifying ductility reduction factors of SDOF systems through a modification factor. Chopra [19] studied the force reduction factors for MDOF systems modeled as shear buildings and their corresponding equivalent SDOF systems. The relative effect of yielding and damping for SDOF systems was also studied. Ayoub and Chenouda [20] developed response spectra plots for inelastic degrading structural systems subjected to seismic excitations.

More recently, Sanchez-Ricart [21] performed a parametric study for moment resisting steel frames designed according to Eurocode 3 and Eurocode 8, considering the design structural overstrength, the ductility, the plastic redistribution, and the strength reduction factor.

Rupakhety and Sigbjörnsson [22] presented ground-motion prediction equations for ductility demand and inelastic spectral displacement of constant-strength perfectly elastoplastic SDOF oscillators. Gillie et al. [23] studied the response of nonlinear SDOF systems to motions with forward directivity (FD). They characterized the response by ductility and period-dependent strength reduction factors. This magnitude dependence was not observed in non-FD motions. Sanchez-Ricart and Plumier [24] reviewed the backgrounds that support the values of the reduction factor in the United States, Europe, and Japan. Ganjavi and Hao [25] studied the seismic response of linear and nonlinear MDOF systems subjected to a group of earthquakes recorded on alluvium and soft soils, considering different shear strength and stiffness distribution patterns.

In spite of the important contributions of the previous studies on the evaluation of the effects of energy dissipation, most of them were limited to SDOF systems, plane shear buildings, or plane moment resisting steel frames. Inelastic behavior and energy dissipation of the structural elements existing in actual three-dimensional systems are not considered. Reyes-Salazar et al. [26], Reyes-Salazar and Haldar [8, 27–29], and Bojórquez et al. [30] found that moment resisting steel plane frames are very efficient in dissipating earthquake-induced energy and that the dissipated energy has an important effect on the structural response. Reyes-Salazar [9] showed that the values of strength reduction factors depend on the amount of dissipated energy, which in turn depends on the plastic mechanism formed in the frames as well as on the loading, unloading, and reloading process at plastic hinges.

As stated earlier, the static equivalent lateral force (SELF) procedure allows for the reduction of the elastic base shear (global response parameter) due, in part, to the structural energy dissipation capacity. The resultant stresses, say axial load or bending moments in columns (local response parameters), are reduced in the same proportion. This procedure is implemented in many codes around the world and is used mainly for plane frames. However, it is important to emphasize that modeling structures as plane frames may not represent their actual behavior due to several reasons: (a) the participation of some structural elements is not considered, (b) the dynamic properties in terms of stiffness, mass distribution, natural frequencies, and energy dissipation characteristics are expected to be different for two-dimensional (2D) and three-dimensional (3D) modeling of such structures, and (c) in the calculation of the axial loads, say in columns, produced by the simultaneous action of the horizontal components, the contribution of each component can be in phase each other for some particular columns but they can be out of phase for some others. It is not possible to consider this situation in the case of the 2D structural representation. Thus, the seismic responses, in terms of global and local parameters, as well as their reduction due to the energy dissipated by viscous damping and yielding of the material, are expected to be different for 3D models and their corresponding simplified representations. Due to the advancements in the computer technology, the computational capabilities have significantly increased in the recent years. It is now possible to estimate the seismic response behavior by modeling structures in three dimensions as complex MDOF systems with thousands of degrees of freedoms and applying the seismic loadings in time domain as realistically as possible. Responses obtained in this way may represent the best estimate of the seismic responses. The accuracy of estimating the effect of the energy dissipated by damping or by yielding of the material on the global and local response parameters by using simplified SDOF or simplified MDOF systems can then be judged by comparing the results with those obtained from the complex 3D formulation.

## 3. Objectives

The specific objectives addressed in this study are as follows. 

*Objective 1.* Estimate the effect of damping on the seismic responses of steel buildings with moment resisting frames (MRF) modeled as 3D systems and compare them with those of the corresponding 2D and SDOF structures. Two cases of 3D models will be considered: (a) with perimeter moment resisting frames (PMRF) and (b) with spatial moment resisting frames (SMRF). The seismic responses are obtained in terms of global (interstory base shear and displacements) and local (axial load and bending moment) parameters. The intensity of the seismic loading is such that no yielding occurs in the models. 

*Objective 2.* Estimate the effect of yielding on the seismic responses of steel buildings with PMRF and SMRF modeled as 3D systems and compare them with those of the corresponding 2D and SDOF structures.

*Objective 3.* Compare the reduction produced by viscous damping with that of yielding of the material.

To reach the objectives of the study, the seismic responses of some structural models are estimated as accurately as possible by using three-dimensional time-history analysis. The models are excited by several time histories recorded at hard and intermediate soils which were selected to represent the different characteristics of strong motions. Energy dissipation and higher mode contributions are explicitly considered. Initially, in order to get elastic behavior, the used earthquake records are scaled down in terms of spectral acceleration in the fundamental mode of vibration of the structure (*S*_*a*_(*T*_1_)); in other words, for a given model, the records are scaled down in such a way that the ordinate values of their pseudoacceleration response spectra for the horizontal component in the N-S direction, evaluated at the fundamental period (*T*_1_) of the model corresponding to N-S direction, are the same for all the records. Then, to get inelastic behavior, the earthquakes are uniformly scaled up in such a way that for the critical earthquake the models develop a level of deformation close to a collapse mechanism or an interstory displacement of about 2.5%. It must be noted that the scaling is different for each structural model since their fundamental periods are different too.

## 4. Mathematical Formulation

In simplified seismic response analysis procedures, like the SELF procedure, the effect of the dissipated energy produced by yielding of the material and by viscous damping on the reduction of the structural response is taken into account by considering an equivalent amount of viscous damping (usually 5% of critical damping). This practice represents a very crude approximation since the nature of the energy dissipation mechanisms in real structures is much more complicated. Among other things, it can be mentioned, for example, that the dissipation of energy depends on the number of plastic hinges formed in the structures and on their duration time. Their location may be continuously changing as loading and unloading occur in the structure. Moreover, in a more realistic consideration of the viscous damping, the damping matrix is expressed as a combination of the mass and stiffness matrix; the latter is continuously changing because of the formation of plastic hinges; hence the damping matrix is continuously changing too. Thus, in order to reasonably estimate the effect of the dissipation of energy, a sophisticated formulation is needed to consider these continuous changes. The intent of this chapter is to briefly present such formulation, which is used in the study.

The estimation of linear and nonlinear seismic responses in time domain for 3D realistic structures is essential to meet the objectives of the study. As will be discussed later, several model steel buildings suggested in the* SAC* steel project [31] will be used for numerical evaluations to address the issues discussed earlier. An assumed stress-based finite element algorithm, developed and implemented by the authors and their associates [32–34] in a computer program, is used to estimate the responses. The procedure and the algorithm have been extensively verified using available theoretical and experimental results [8, 29]. The procedure estimates the responses by considering the main sources of energy dissipation and material and geometry nonlinearities. In this approach, an explicit form of the tangent stiffness matrix is derived without any numerical integration. Fewer elements can be used in describing a large deformation configuration without sacrificing any accuracy, and the material nonlinearity can be incorporated without losing its basic simplicity. It gives very accurate results and is very efficient compared to the commonly used displacement-based approach. Only the basic equations are given, but the complete description of the algorithm can be found in the literature [33–35].

The nonlinear dynamic equation of motion that governs the problem under consideration can be expressed as follows:

(1) m(t+Δt)U¨(k)+C(t+Δt)tU˙(k)+K(t+Δt)tΔU(k)  =F(k)(t+Δt)−R(k−1)(t+Δt)−mU¨g(k).

The nonlinear iterative strategy is used to solve the equation where **m**, **C**, and **K** are the mass, damping, and the tangent stiffness matrixes, respectively. $\ddot{U}$ and $\dot{U}$ are the acceleration and velocity vectors, respectively, Δ**U** is the incremental displacement vector, **F** is the external load vector, **R** is the internal force vector, and ${\ddot{U}}_{g}$ is the ground acceleration vector. The superscripts *t*, Δ*t*, and *k* represent the time, time increment, and the iteration number, respectively. Rayleigh-type damping is commonly used for nonlinear analysis in the profession since it is a function of the mass and the tangent stiffness matrices which represents the current state of deformation of a structure and it is considered in this study. Thus, the damping matrix is continuously updated by using the mass and tangent stiffness matrices.

The mass matrix is assumed to be concentrated type. The step-by-step direct integration numerical analysis procedure and the Newmark *β* method [32] are used to solve the nonlinear seismic governing equation of the problem. Explicit expressions for the tangent stiffness matrix and the internal force vector are developed for each beam-column element using the assumed stress-based finite element method for the* k*th iteration at time *t*. The nonlinear elastic tangent stiffness matrix for a beam-column element, **K** ^*e*^, can be represented as

(2) $$\begin{array}{c} K^{e}=A_{\sigma do}^{T}A_{\sigma \sigma }^{-1}A_{\sigma do}+A_{ddo}, \end{array}$$

where **A**_*σσ*_^−1^ is the elastic property matrix, **A**_*σdo*_ is the transformation matrix, and **A**_*ddo*_ is the geometric stiffness matrix. Similarly, the internal force vector of an element level, **R** ^*e*^, can be expressed as

(3) $$\begin{array}{c} R^{e}=-A_{\sigma do}^{T}A_{\sigma \sigma }^{-1}R_{\sigma }+R_{do}, \end{array}$$

where **R**_*do*_ is the homogeneous part of the internal force vector and **R**_*σ*_ is the deformation difference vector. The explicit expressions for all the terms in (2) and (3) can be found in the literature [33, 36].

The structural behavior discussed above also needs to be modified to consider material nonlinearity. In this study, the material is considered to be linear elastic except at plastic hinges. Concentrated plasticity behavior is assumed at plastic hinge locations. For mathematical modeling, plastic hinges are assumed to occur at locations where the combined action of axial force, torsion, and bending moments satisfies a prescribed yield function. This is discussed in detail elsewhere [37]. The yield function for three-dimensional beam-column elements and W-shape sections (used in this study) has the following form:

(4) $$\begin{array}{c} {\left(\frac{P}{P_{n}}\right)}^{2}+{\left(\frac{M_{X}}{M_{nX}}\right)}^{2}+{\left(\frac{M_{Y}}{M_{nY}}\right)}^{2}+{\left(\frac{M_{Z}}{M_{nZ}}\right)}^{2}-1=0, \end{array}$$

where *P* is the acting axial force, *M*_*X*_ and *M*_*Y*_ are the acting bending moments with respect to the major and minor axis, respectively, *M*_*Z*_ is the acting torsional moment, *P*_*n*_ is the axial strength, *M*_*nX*_ and *M*_*nY*_ are the flexural strength with respect to the major and minor axis, respectively, and *M*_*nZ*_ is the torsional strength. The presence of plastic hinges in the structure will produce additional axial deformation and relative rotation in a particular element. Thus, the tangent stiffness matrix needs to be modified if plastic hinges form. The elastoplastic tangent stiffness matrix **K**_*P*_^*e*^ and the elastoplastic internal force vector **R**_*P*_^*e*^ can be obtained by modifying the corresponding elastic matrixes as follows [34, 35]:

(5) $$\begin{array}{c} K_{P}^{e}=K^{e}-A_{\sigma do}^{T}A_{\sigma \sigma }^{-1}V_{P}C_{P}^{T}A_{\sigma do}\\ R_{P}^{e}=A_{\sigma do}^{T}\left(A_{\sigma \sigma }^{-1}V_{P}C_{P}^{T}-A_{\sigma \sigma }^{-1}\right){\hat{R}}_{\sigma }+R_{do}. \end{array}$$

As stated earlier, one part of the input energy imposed on a structure during seismic loading is dissipated by viscous damping (*E*_*D*_) and by the hysteretic (yielding) behavior of the material (*E*_*P*_) at plastic hinges. *E*_*P*_ is equivalent to the work done by the resultant stresses through the corresponding plastic deformations which can be expressed as

(6) $$\begin{array}{c} E_{P}=\;\overset{n}{\underset{i=1}{\sum }}M_{PX}\;\theta_{PX}+\overset{n}{\underset{i=1}{\sum }}M_{PY}\;\theta_{PY}+\overset{n}{\underset{i=1}{\sum }}P_{P}\;H_{P}, \end{array}$$

where *n* is the number of plastic hinges developed, *M*_*PX*_ and *M*_*PY*_ are the bending moments with respect to the major and minor axes, respectively, *P*_*P*_ is the axial load, acting on a plastic hinge, and *θ*_*PX*_, *θ*_*PY*_, and *H*_*P*_ are the corresponding plastic rotations and axial deformation.

The energy dissipated by viscous damping is estimated as

(7) $$\begin{array}{c} E_{D}=\overset{{\dot{U}}_{2}}{\underset{{\dot{U}}_{1}}{\int }}C\dot{U}\;du=\overset{t_{2}}{\underset{t_{1}}{\int }}U^{T}C\dot{U}\;dt, \end{array}$$

where *t*_1_ and *t*_2_ define a generic time interval and ${\dot{U}}_{1}$ and ${\dot{U}}_{2}$ are the vectors of velocities at the beginning and at the end of the interval.

In order to estimate the individual effect of the energy dissipated by damping on the seismic response, elastic behavior of the structural models is considered. The structural responses are estimated considering 0%, 2%, 5%, and 10% of critical damping (*ζ* = 0,2, 5 and 10%); then the damping effect is estimated as

(8) $$\begin{array}{c} R_{\zeta }=\frac{\text{Response}\left(\zeta =2\%\right)}{\text{Response}\left(\zeta =0\%\right)},\;\;R_{\zeta }=\frac{\text{Response}\left(\zeta =5\%\right)}{\text{Response}\left(\zeta =2\%\right)}\\ \text{or}\;R_{\zeta }=\frac{\text{Response}\left(\zeta =10\%\right)}{\text{Response}\left(\zeta =5\%\right)}, \end{array}$$

where the damping reduction factor, *R*_*ζ*_, represents the reduction of the response when damping is changed from 0 to 2%, from 2 to 5%, or from 5 to 10%. These ranges of damping will be referred to hereafter as 0–2, 2–5, and 5–10 ranges, respectively. It is important to mention that even though *ζ* = 10% is used, it is unrealistic for inherent damping. However, it can be related to structures with added damping (damping systems). A significant amount of information about this topic can be found in the literature [10–12, 38, 39].

In order to estimate the reduction in the response produced only by the energy dissipated by yielding of the material, for a given amount of damping, the elastic and inelastic responses are compared. 2%, 5%, and 10% of critical damping are considered. The yielding reduction factor is estimated as

(9) $$\begin{array}{c} R_{P}=\frac{\text{Inelastic}\;\text{response}\left(\zeta =2\%\right)}{\text{Elastic}\;\text{response}\left(\zeta =2\%\right)},\\ R_{P}=\frac{\text{Inelastic}\;\text{response}\left(\zeta =5\%\right)}{\text{Elastic}\;\text{response}\left(\zeta =5\%\right)},\\ \text{or}\;R_{P}=\frac{\text{Inelastic}\;\text{response}\left(\zeta =10\%\right)}{\text{Elastic}\;\text{response}\left(\zeta =10\%\right)}. \end{array}$$

As it will be shown later, additional subscripts are added to *R*_*ζ*_ and *R*_*P*_ to differentiate global from local response parameters or one structural representation from another.

## 5. Structural Models

### 5.1. 3D Buildings with PMRF (SAC Models)

As part of the SAC steel project [31], several steel model buildings were designed by three consulting firms. They considered 3-, 10-, and 22-level buildings. These buildings are supposed to satisfy all code requirements existing at the time of the project development for the following three cities: Los Angeles (Uniform Building Code, 1997) [40], Seattle (Uniform Building Code, 1997) [40], and Boston (Building Officials & Code Administration (BOCA, 1993)) [41]. The 3- and 10-level buildings located in the Los Angeles area are considered in this study for numerical evaluations to address the issues discussed earlier. They will be denoted hereafter by Models SC1 and SC2, respectively, and, in general, they will be referred to as the SAC Models. These models have been used in many investigations.

**Figure 1:**
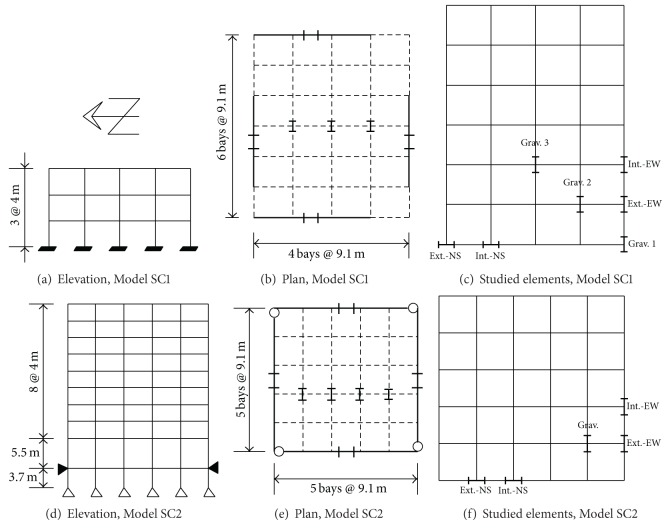
Elevation, plan, and element location for Models SC1 and SC2.

The elevations of the models are given in Figures 1(a) and 1(d) and their plans are given in Figures 1(b) and 1(e), respectively. They will be denoted hereafter by Models SC1 and SC2, respectively. The fundamental periods of Model SC1 are estimated to be 1.03, 0.99, and 0.07 sec., in the N-S (horizontal), W-E (horizontal), and *Z* (vertical) directions, respectively. The corresponding values for Model SC2 are 2.22, 2.11, and 0.16 sec. The 10-level building has a single-level basement. The columns of the PMRF of Model SC1 are fixed at the base while those of Model SC2 are pinned, as considered in the FEMA report [31]. In all these frames, the columns are made of steel Grade-50 and the girders are of A36 steel. For both models, the columns in the gravity frames (GF) are considered to be pinned at the base. All the columns in the PMRF bend about the strong axis and the strong axes of the gravity columns are oriented in the N-S direction, as indicated in Figures 1(b) and 1(e). The particular elements to study the response in terms of local responses parameters are given in Figures 1(c) and 1(f) for Models SC1 and SC2, respectively. In these figures, the PMRF are represented by continuous lines while the interior GF are represented by dashed lines. For Model SC2, the PMRF meet at a corner. In this case, the beam-to-column connections are considered to be pinned to eliminate weak-axis bending (Figure 1(e)). As it can be seen, the buildings are essentially symmetrical in plan; thus no significant torsional moments are expected to occur. Sizes of beams and columns, as reported, are given in Table 1 for the two models. The designs of the PMRF in the two orthogonal directions were practically the same. Additional information for the models can be obtained from the FEMA report [31].

The buildings are modeled as complex MDOF systems. Each column is represented by one element and each girder of the PMRF is represented by two elements, having a node at the midspan. The slab is modeled by near-rigid struts, as considered in the FEMA study. Each node is considered to have six degrees of freedom when the buildings are modeled in three dimensions. It is worth mentioning that even though the buildings are modeled as complex 3D MDOF structures, many simplifications still remain since the contribution of the slab in the bending capacity of beams, stiffness of connection of interior gravity frames, flexibility of the beam-to-column connection of moment resisting frames, contribution of the panel, and column base flexibility are not considered.

### 5.2. 3D Buildings with SMRF (EQ Models)

Because of economic considerations and the fragility of weak-axis connections, the standard practice during the recent past (after the 80s) in USA has been to build steel buildings with fully restrained connections (FRC) only on two frame lines in each direction. The redundancy of the buildings, however, is tremendously reduced. In Mexico, it is common to use steel buildings with FRC at the perimeter and the interior, in both horizontal directions. Due to the large number of FRC of this system, its redundancy is expected to be greater than those of the systems with only PMRF although the structural analysis is more complicated. Comparison of the performance of these two structural systems under the action of severe seismic loads, in terms of the effect of energy dissipated by damping and yielding, is undoubtedly of great interest to the profession and therefore it is addressed in this study. Equivalent models with SMRF are considered for this purpose. The equivalent models are designed in such a way that their elastic fundamental period, total mass, yield strength, and lateral stiffness are fairly the same as those of the corresponding buildings with PMRF.

The member properties of the equivalent buildings are selected for one direction, say the N-S directions, and then in order to keep the equivalence, the same properties are assigned to the other direction. They are selected by considering the beam and column properties of the PMRF oriented in the direction under consideration, in addition to those of the beams and columns of the perpendicular PMRF. It must be noted that the columns of the later frames bend with respect to their minor axes. The ratio of moments of inertia, or plastic moments, between beams and columns was tried to keep as close as possible for the two structural systems. The same was considered for the case of interior and exterior columns. The equivalent models are referred to, in particular, as Models EQ1 and EQ2 for the 3- and 10-level buildings, respectively, and, in general, as EQ Models. The resulting sections are shown in Table 2.

### 5.3. 2D Models

For seismic analysis and design purposes, steel buildings with PMRF are modeled as plane frames. In this process, it is assumed that, for a given horizontal direction, half of the seismic loading is supported by the two PMRF oriented in that direction. However, as stated earlier, modeling 3D buildings as plane frames may not represent the actual behavior of the structure since the participation of some elements is not considered. Moreover, the stiffness and the dynamic properties in terms of natural frequencies, damping or energy dissipation characteristics, are expected to be different for two-dimensional and three-dimensional modeling of such structures. Thus, it will be of interest to estimate the relative effect of damping and yielding on the seismic response of steel buildings with PMRF, modeled as 3D structural systems, and compare it with that of the structures modeled as 2D systems. These models will be denoted by Models 2D1 and 2D2 for the 3- and 10-level models, respectively, and, in general, as 2D models.

### 5.4. SDOF Models

**Figure 2:**
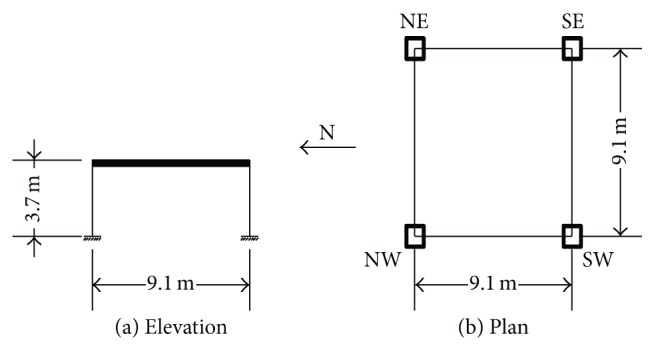
Elevation and plan of the equivalent SDF models.

The relative effect of damping and yielding is also studied for* equivalent *single degree of freedom (SDOF) systems. One equivalent SDOF model is considered for the 3- and 10-level buildings. They will be particularly denoted hereafter by Models SD1 and SD2, respectively, and as SDF models in general. These systems have a SDOF in each horizontal direction. The elevation and plan of these systems are shown in Figure 2. The weight of the* equivalent* SDOF system is the same as the total weight of its corresponding MDOF system and its lateral stiffness is selected in such a way that its natural period is the same as the fundamental natural period of its corresponding MDOF system. In order to have the equivalence in both horizontal directions, square hollow structural sections were used for columns. They were HSS26×26×1/2 and HSS22×22×1/2 for the 3- and 10-level models, respectively. The damping ratio and the yielding strength are selected to be the same for the SAC and the SDF models. The latter was determined from a pushover analysis. It must be noted that, in a strict sense, the simpler models are not the typical SDOF systems studied in the structural dynamics textbooks since axial forces can be developed in the columns under the action of horizontal excitations.

### 5.5. Earthquake Loading

**Figure 3:**
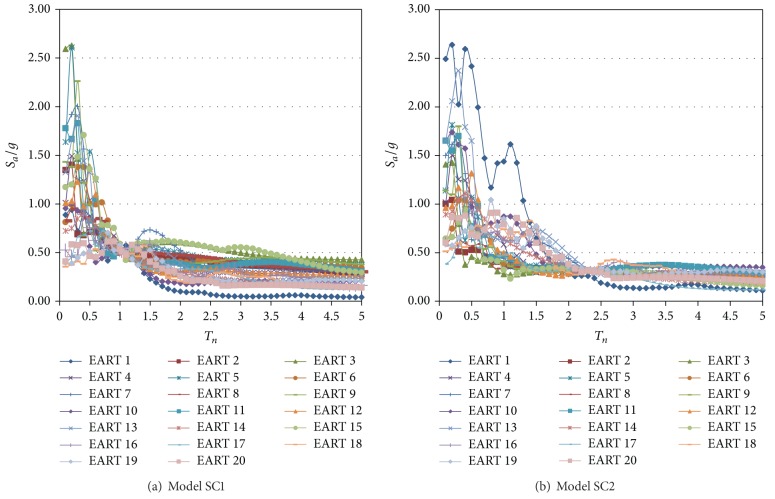
Elastic response spectra for scaled earthquakes.

Dynamic responses of a structure excited by different earthquake time histories, even when they are normalized in terms of *S*_*a*_(*T*_1_) or in terms of the peak ground acceleration, are expected to be different, reflecting their different frequency contents. Thus, evaluating structural responses excited by an earthquake may not reflect the behavior properly. To study the responses of the models comprehensively and to make meaningful conclusions, they are excited by twenty recorded earthquake motions in time domain with different frequency contents, recorded at different locations. The models are simultaneously subjected to the action of the three components of the earthquake records for the case of three-dimensional and SDOF models, and to one horizontal component at a time and the vertical component, for the case of the plane models. As stated earlier, the earthquake records are scaled in terms of spectral acceleration in the fundamental mode of vibration of the structure (*S*_*a*_(*T*_1_)) first for elastic behavior and then for a significant level of deformation. The characteristics of these earthquake time histories are given in Table 3. They were selected to cover a wide range of excitation frequency content, to have PGAs close to or larger than 0.20 g, and to have a strong phase duration of at least 30 seconds with an acceleration close to or larger than 0.15 g. In the table, the symbols *T*, EP, DE, and MA denote the predominant period, the distance to the epicenter, and the depth and the magnitude of the earthquake, respectively. The peak ground acceleration, velocity, and displacement (PGA, PGV, and PGD) are also observed. As shown in the table, the predominant periods of the earthquakes for the N-S component vary from 0.12 to 0.88 sec. The predominant period for each earthquake is defined as the period where the largest peak in the elastic response spectrum occurs, in terms of pseudoaccelerations. The response spectra for the N-S component for 5% damping for a significant level of deformation are given in Figure 3. The earthquake time histories were obtained from the data sets of the National Strong Motion Program (NSMP) of the United States Geological Surveys (USGS). Additional information on these earthquakes can be obtained from this database.

## 6. Objective 1. Effect of Damping

### 6.1. The SAC Models

**Figure 4:**
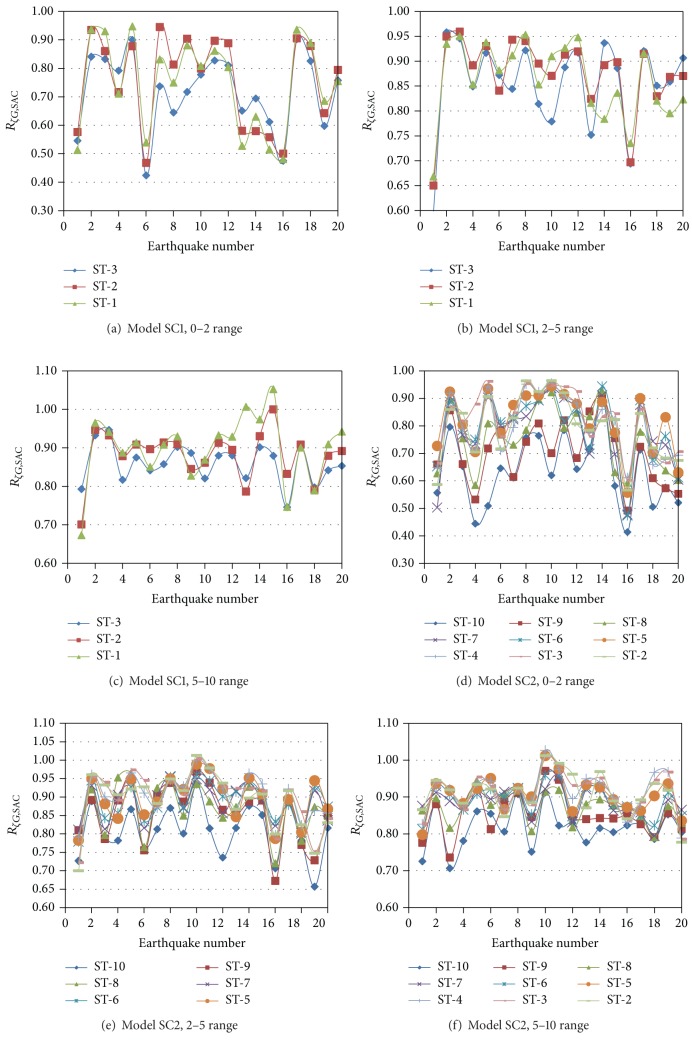
Global damping reduction factors for shear, SAC Models, and N-S direction.

The symbol *R*_*ζG*,SAC_ is specifically used to represent the damping reduction factors for global response parameters of the SAC Models. For a given model, earthquake, direction and interstory, the damping reduction factors for shears or displacements are estimated and averaged over all the plane frames that conform the 3D structure for the interstory under consideration. Results for interstory shears are presented in Figure 4 for Models SC1 and SC2 and the N-S direction. In this figure, the word “ST” stands for the story level. It can be observed that the *R*_*ζG*,SAC_ values significantly vary from one earthquake to another, even though the earthquakes were normalized with respect to *S*_*a*_(*T*_1_). It reflects the effect of the earthquake frequency contents and the contribution of several modes on the structural responses. Values closer to 0.4 are observed in many cases for the 0–2 damping range indicating that increasing damping from 0 to 2% can reduce the response in almost 60%. It is also noted that the reduction in the response is, in general, larger for the 0–2 than for the 2–5 range which in turn is larger than that of the 5–10 range, confirming the well-known results observed in typical SDOF systems: damping is more effective in reducing the response in low ranges. Results also indicate that the reduction in the response is larger for the upper interstory. Plots for *R*_*ζG*,SAC_ for the E-W direction were also developed but are not shown. The major conclusion made before applies to this case. The only additional observation that can be made is that, for both horizontal directions, the variation of *R*_*ζG*,SAC_ from one story to another generally decreases as damping increases. The effect of damping on the reduction of the average interstory displacements is also estimated; considering two models, two directions, and three cases of damping increments, as for the case of average interstory shears, 12 figures were developed, but they are not shown. A high correlation is observed between the plots of interstory shears and displacements. Thus, the major conclusions made before are valid for the displacement reduction.

**Figure 5:**
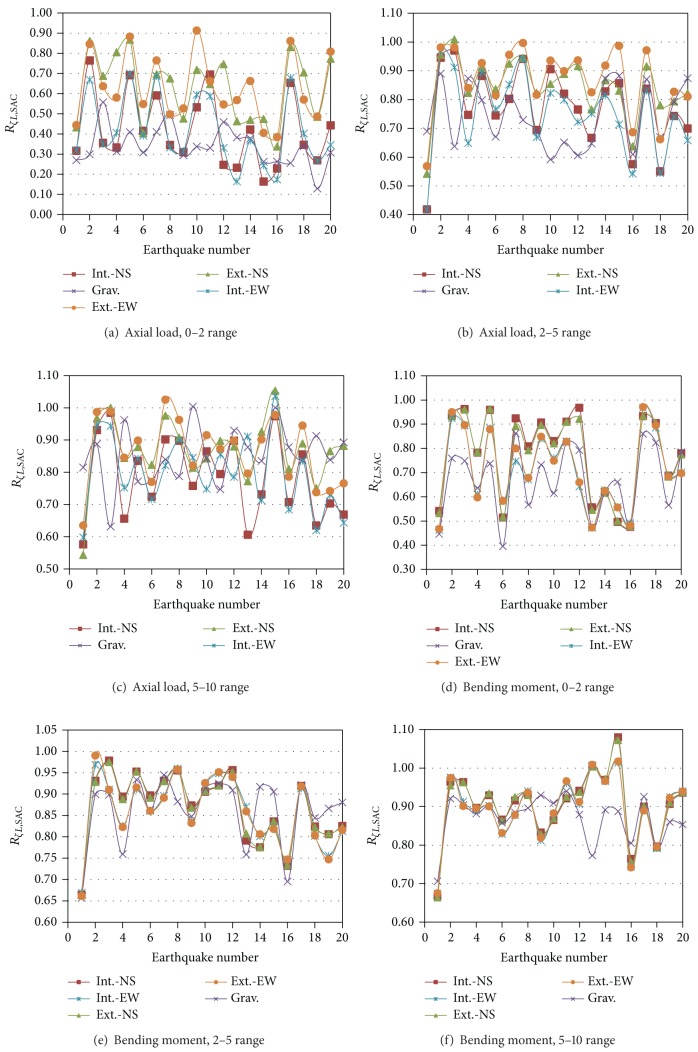
Local damping reduction factor for element forces, Model SC1.

The damping reduction factors for local response parameters (*R*_*ζL*,SAC_) are considered next. Typical values of *R*_*ζL*,SAC_ for axial loads and bending moments on selected members (Figure 1(c)) of Model SC1 are given in Figure 5. The results are similar in one sense to those of global response parameters but different in another: the *R*_*ζL*,SAC_ values significantly vary from one earthquake to another and from one interstory to another; the damping reduction factors, however, seem to be smaller for local response parameters, particularly for axial loads; values lower than 0.20 are observed in some cases for the 0–2 damping range, implying a response reduction larger than 80%. The variation of *R*_*ζL*,SAC_ from one column to another generally decreases as damping increases and it is smaller for bending moment than for axial loads.

As commented above, most of the values of *R*_*ζL*,SAC_ (Figure 5) are smaller than unity implying that the response decreases as damping increases. For some cases, however, the values are slightly larger than unity implying that the response increases with an increment of damping, contradicting the results of typical SDOF system. The reason for this is, as stated earlier, that the dynamic properties in terms of stiffness, natural frequencies, viscous damping, and energy dissipation characteristics, as well as loading conditions of complex 3D systems, are quite different from those of typical SDOF systems and consequently their responses are expected to be different too. Moreover, a change in damping produces a change in the phase of the response of each mode allowing the possibility of larger responses.

### 6.2. The EQ Models

**Figure 6:**
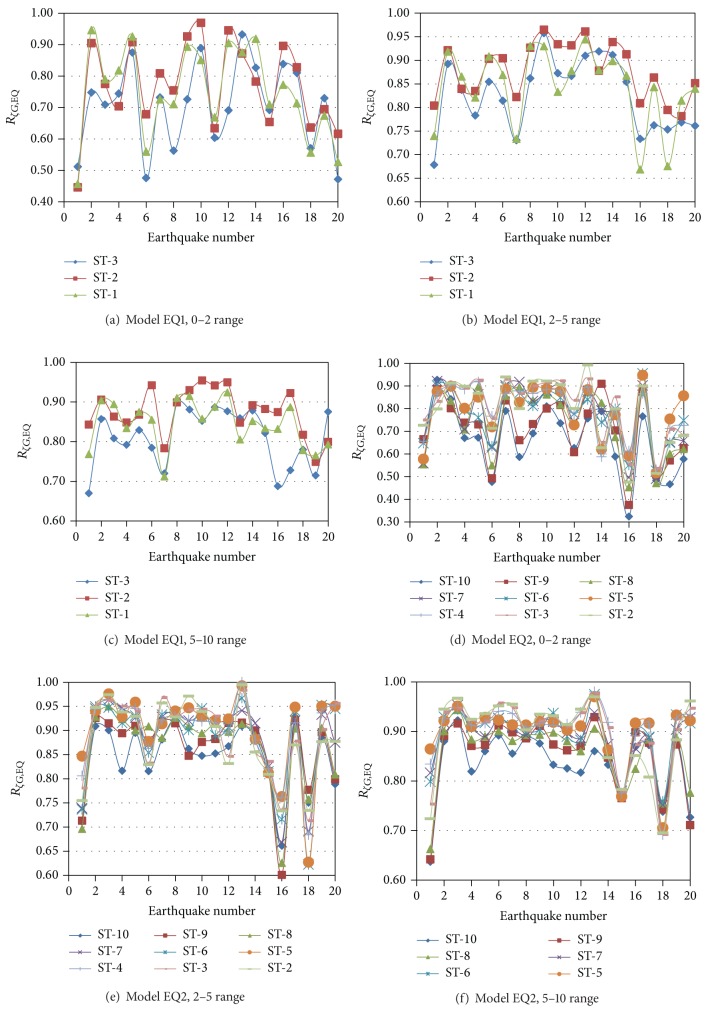
Global damping reduction factor for shear, EQ Models, and N-S direction.

**Figure 7:**
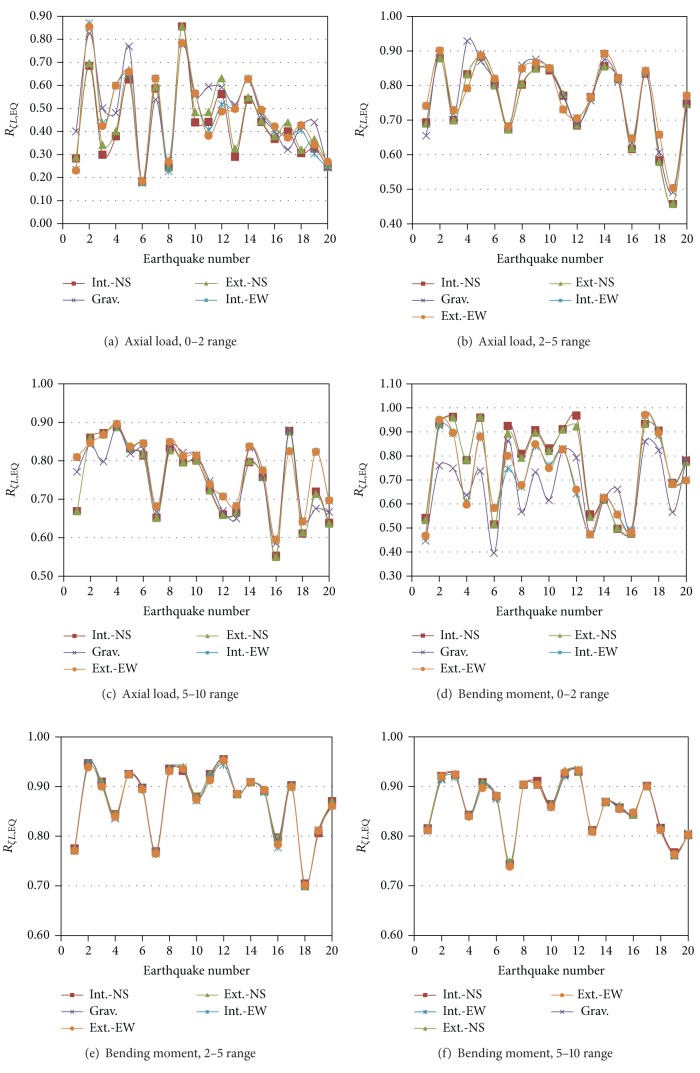
Local damping reduction factor for element forces, Model EQ1.

The *R*_*ζG*,EQ_ parameter is used to represent the global damping reduction factors for the equivalent (EQ) 3D models. The results for interstory shears are presented in Figure 6 for Models EQ1 and EQ2 and the N-S direction. As for the 3D models with PMRF (SAC Models), the reduction factors significantly vary from one earthquake to another and from one story to another reflecting the effect of earthquake frequency contents and the contribution of several modes of vibrations. From a comparison of all the plots, it is noted that the major observations made for the SAC Models also apply the EQ Models; the only additional observation that can be made is that the reduction values are slightly larger for the SAC Models. The local damping reduction factors for the EQ Models (*R*_*ζL*,EQ_) for both axial loads and bending moments are given in Figure 7 for Model EQ1 and the N-S direction. The results resemble those of the SAC Models in the sense that the reduction of the response is larger for local than for global parameters, larger for axial loads than for bending moments, and that the variation of the reduction factors from one column to another, which decreases as damping increases, is smaller for bending moment than for axial load. For a given earthquake, the bending moment reductions, for the 2–5 or 5–10 range, are essentially the same for all the columns under consideration.

### 6.3. The 2D and SDOF Models

The global (*R*_*ζG*,2D_) and local (*R*_*ζL*,2D_) damping reduction factors, for shears and displacements, of the buildings modeled as plane structures, as well as those of the buildings modeled as SDOF systems (*R*_*ζG*,SDF_ and *R*_*ζl*,SDF_), are also calculated. The corresponding plots are not presented because there are no significant differences between these results and those of the SAC or the EQ Models. It can be commented, however, that, in general, as for the SAC and EQ Models, the reduction factors are larger for global than for local parameters, particularly for the case of axial load, and that the variation of the reduction factors from one structural element to another (base columns) is larger for axial loads than for bending moments.

### 6.4. Results in terms of Statistics

The global reduction factors of the SAC, EQ, and 2D models are averaged over all the stories and then their statistics are estimated over all the earthquakes. For the case of the SDF models, their statistics are estimated over all the earthquakes. The results for the 0–2 range are given in Tables 4 and 6 for shear and displacements, respectively, while the corresponding results for the 2–5 range are given in Tables 5 and 7. The statistics for the 5–10 range are not presented. It is observed that the mean values are quite similar for the SAC and the EQ Models for the case of shears and the 0–2 range; they vary from 0.70 to 0.91. The largest and smallest values observed are for the SDF and the 2D models, respectively. The uncertainty in the estimation is moderate; the coefficient of variation ranges from 0.11 to 0.22. For the case of shears and 2–5 range, the mean reduction factors are quite similar for the four structural representations, which in turn are larger (implying a smaller shear reduction) than those of the 0–2 range. The uncertainty in the estimation is, however, much smaller for the 2–5 than for the 0–2 range. From a comparison of the mean values of the reduction factors for shear and displacements, it is observed that the mean and the COV values are quite similar, indicating a high correlation between these two parameters.

The statistics for local response parameters are given in Tables 8 and 9 for the 0–2 and 2–5 range, respectively. The variation of the mean reduction factors for the 0–2 range from one structural representation to another, from one model to another, from one response parameter to another, or from one direction to another is larger for local than for global response parameters. The minimum observed value (greater response reduction) is observed to be 0.35 for axial load at interior columns of the N-S direction of the 3-level 2D model while the largest one (minimum response reduction) is 0.91 for bending moment at interior column of the SDOF model of the 10-level building. The most important observation that can be made is that the reduction factors can be significantly smaller for local than for global response parameters, as concluded before from particular figures. On the other hand, the uncertainty in the estimation of the reduction factors may be significantly larger for local response parameters, particularly for the case of the 0–2 range.

## 7. Objective 2. Effect of Yielding

As commented in Section 3, the different earthquake acceleration records are first normalized with respect to the pseudoacceleration evaluated at the fundamental structural period (*S*_*a*_(*T*_1_)) and then they are uniformly scaled up in such a way that considerable yielding occurs in the models for the critical earthquake. A level of deformation close to a collapse mechanism or an interstory displacement of about 2.5% was developed. The maximum interstory displacement occurred for the 2D models; the drifts were smaller for the SAC and EQ Models. It was observed that about 6 to 23 plastic hinges were formed in the cases where yielding occurred. As for the case of damping reduction factors, 16 figures were developed for yielding reduction factors, for each structural representation; however, only the statistics for interstory shears will be presented. The statistics for the global yielding reduction factors (*R*_PG_) are first discussed. They are given in Tables 10 and 11 for *ζ* = 2% and *ζ* = 5%, respectively. It is observed that, for a given structural representation, the reduction factors can significantly vary from one earthquake to another without showing any trend; they vary from 0.71 to 1.15, from 0.65 to 1.06, and from 0.50 to 0.91 for the SAC, EQ, and 2D models, respectively. For the case of equivalent SDF models in which yielding was not significant, the reduction factors resulted to be close to unity practically in all cases. From the individual and the mean values of *R*_PG_, it is observed that the reduction is about 20% larger for the 2D models than for the SAC or the EQ Models. The uncertainty in the estimation is small in all the cases and it is slightly larger for 5% than for 2% damping.

The statistics for the local yielding reduction factors are given in Tables 12 and 13 for *ζ* = 2% and *ζ* = 5%, respectively. It is observed that they are smaller than yielding reduction factors of global response parameters. They can significantly vary from one structural representation to another and from one response parameter to another. The values resulted smaller for axial loads than for bending moments and smaller for the EQ than for the other structural representations. The minimum observed reduction factors were for the axial loads of the EQ Models, which range from 0.17 to 0.25 for the case of 5% damping, while the maximum observed reduction factors were for bending moments of the SAC Models for 5% damping which range from 0.92 to 1.19. The uncertainty in the estimation is larger for local than for global yielding reduction factors. Thus, global yielding reduction factors are smaller than local yielding reduction factors which in turn depend on the particular force and the location under consideration, reflecting the limitations of the commonly used static equivalent lateral force (SELF) procedure where local and global response parameters are reduced in the same proportion.

## 8. Objective 3. Comparison of the Reduction Factors for Viscous Damping and Yielding of the Material

From a comparison of the damping and yielding global reduction factors, it is observed that, excepting those of the 2D models, they are smaller for damping implying a larger reduction in the global structural response. The uncertainty in the estimation is, excepting that of the SDF models, larger for the case of yielding. For local reduction factors, they are smaller for the case of yielding and axial loads; for bending moments, however, they resulted to be smaller for damping. For both cases the uncertainty in the estimation can be considerable.

As stated earlier, in the estimation of both, damping and yielding reduction factors, in order to have the same participation of the fundamental structural mode, the different earthquake acceleration records were first normalized with respect to the pseudoacceleration evaluated at the fundamental structural period (*S*_*a*_(*T*_1_)) and then they were uniformly scaled up in such a way that for the critical earthquake considerable yielding occurred. The maximum interstory displacement developed was for the 2D models for some earthquakes. However, yielding was not significant for many of the earthquakes even for the case of 2D models and consequently the yielding reduction factors do not represent the maximum ones that could have been developed in the models. Thus, the conclusion made in relation to the yielding of the material is for the particular level of structural deformation and could significantly change if more yielding is allowed in the structures. For those cases where considerable yielding occurred in the models (as for the 2D models), the global or local reduction factors are comparable and even much larger than those of damping.

## 9. Conclusions

The evaluation of the effect of viscous damping and yielding of the material on the reduction of the seismic responses of steel buildings with perimeter moment resisting frames, modeled as three-dimensional (3D) complex multidegree of freedom (MDOF) systems, constitutes the main objective of this paper. The results are compared with those of equivalent 3D structural representations with spatial moment resisting frames as well as with those of bidimensional and equivalent single degree of freedom idealizations. Two steel model buildings subjected to twenty recorded strong motions, scaled in terms of the spectral acceleration at the fundamental mode of vibration of the structures (*S*_*a*_(*T*_1_)), are used in the study. The effects are expressed in terms of global and local damping reduction factors and in terms of global and local yielding reduction factors. The results indicate that the magnitude of the response reduction significantly varies from one earthquake to another, even though the earthquakes were normalized with respect to the same pseudoacceleration, reflecting the influence of the earthquake frequency contents and the contribution of several vibration modes to the structural responses. It is also observed that the reduction in the response produced by damping may be larger or smaller than that of yielding. This reduction can significantly vary from one structural representation to another and is smaller for global than for local response parameters, which in turn depends on the particular local response parameter and the location of the structural element under consideration. It reflects the limitations of the commonly used static equivalent lateral force (SELF) procedure where local and global response parameters are reduced in the same proportion. The reason for this is that the dynamic properties in terms of stiffness, natural frequencies, viscous damping, and energy dissipation characteristics as well as loading conditions of complex 3D systems are quite different from those of simplified bidimensional or SDOF idealizations; consequently their responses are expected to be different too. The uncertainty in the estimation is significantly larger for local than for global response parameters and decreases as damping increases. It is also noted that the reduction in the response is, in general, larger for low ranges of damping confirming what is observed from the results of typical SDOF systems. Based on the results of this study, it is concluded that estimating the effect of viscous damping and yielding of the material on the seismic response of steel buildings by using simplified models may be a very crude approximation. Moreover, because of the significant differences between the damping and yielding reductions, the effect of yielding should explicitly be calculated by using complex 3D MDOF models instead of estimating it in terms of equivalent viscous damping. The findings of this paper are for the particular structural systems, models, and earthquakes used in the study. Much more research is needed to reach more general conclusions.

## Figures and Tables

**Table 1:** Beam and columns sections for the SAC models.

Model	Moment resisting frames				Gravity frames		
	Story	Columns		Girder	Columns		Beams
		Exterior	Interior		Below penthouse	Others	
1	1\2	W14 × 257	W14 × 311	W33 × 118	W14 × 82	W14 × 68	W18 × 35
	2\3	W14 × 257	W14 × 312	W30 × 116	W14 × 82	W14 × 68	W18 × 35
	3\roof	W14 × 257	W14 × 313	W24 × 68	W14 × 82	W14 × 68	W16 × 26

2	−1/1	W14 × 370	W14 × 500	W36 × 160	W14 × 211	W14 × 193	W18 × 44
	1/2	W14 × 370	W14 × 500	W36 × 160	W14 × 211	W14 × 193	W18 × 35
	2/3	W14 × 370	W14 × 500, W14 × 455	W36 × 160	W14 × 211, W14 × 159	W14 × 193, W14 × 145	W18 × 35
	3/4	W14 × 370	W14 × 455	W36 × 135	W14 × 159	W14 × 145	W18 × 35
	4/5	W14 × 370, W14 × 283	W14 × 455, W14 × 370	W36 × 135	W14 × 159, W14 × 120	W14 × 145, W14 × 109	W18 × 35
	5/6	W14 × 283	W14 × 370	W36 × 135	W14 × 120	W14 × 109	W18 × 35
	6/7	W14 × 283, W14 × 257	W14 × 370, W14 × 283	W36 × 135	W14 × 120, W14 × 90	W14 × 109, W14 × 82	W18 × 35
	7/8	W14 × 257	W14 × 283	W30 × 99	W14 × 90	W14 × 82	W18 × 35
	8/9	W14 × 257, W14 × 233	W14 × 283, W14 × 257	W27 × 84	W14 × 90, W14 × 61	W14 × 82, W14 × 48	W18 × 35
	9/roof	W14 × 233	W14 × 257	W24 × 68	W14 × 61	W14 × 48	W16 × 26

**Table 2:** Beam and columns sections for the equivalent models.

Model	Story	Columns		Girders
		Exterior	Interior	
3-level	1\2	W16 × 67	W14 × 109	W12 × 170
	2\3	W16 × 67	W14 × 109	W14 × 120
	3\roof	W16 × 67	W14 × 109	W16 × 40

10-level	−1/1	W18 × 143	W21 × 166	W24 × 162
	1/2	W18 × 143	W21 × 166	W24 × 162
	2/3	W18 × 143	W21 × 166	W24 × 162
	3/4	W18 × 143	W21 × 147	W21 × 166
	4/5	W18 × 143	W21 × 147	W21 × 166
	5/6	W21 × 93	W27 × 84	W21 × 166
	6/7	W21 × 93	W27 × 84	W21 × 166
	7/8	W14 × 145	W18 × 106	W24 × 68
	8/9	W14 × 145	W18 × 106	W12 × 152
	9/roof	W24 × 62	W18 × 97	W16 × 67

**Table 3:** Earthquake records, N-S component.

Number	Event	Date	Station	*T* (sec)	EP (km)	DE (km)	MA	PGA (cm/sec²)	PGV (cm/s)	PGD (cm)
1	Mexicali, Baja California, Mexico	06/09/1980	Cerro Prieto	0.12	20	4	6.30	308	19.50	6.15

2	Lake Elsinore, California	09/02/2007	Lake Mathews Dam	0.15	13	12	4.70	507	6.60	0.22

3	Obsidian Butte, California	09/02/2005	Salton Sea Wildlife Refuge	0.19	2	9	4.84	236	12.00	3.93

4	Pinnacles, California	08/27/2011	Bear Valley, Webb Residence	0.21	13	7	4.62	239	5.88	0.37

5	Pinnacles, California	04/06/2012	Paicines, Hain Homestead	0.23	3.8	5	4.00	232	4.45	0.18

6	Parkfield, California	09/28/2004	Parkfield, Eades	0.24	9.8	7	6.00	384	25.80	7.34

7	Yucaipa, California	06/16/2005	Redlands, Seven Oaks Dam	0.25	10	11	5.50	290	6.30	0.26

8	Guadalupe Victoria, BC, Mexico	12/30/2009	Holtville	0.26	42	6	5.80	322	25.70	5.28

9	Chatsworth, California	08/09/2007	Granada Hills, Porter Ranch	0.27	6	7	4.60	148	4.77	0.34

10	Lennox, California	05/18/2009	Compton, Cressey Park	0.30	9	15	4.65	207	6.84	0.67

11	Anza, California	06/12/2005	Mountain Center, Pine Meadows R.	0.31	5	14	5.20	200	9.38	0.86

12	Cobb, California	02/18/2004	Cobb	0.32	2	3	4.40	213	9.00	0.48

13	Alum Rock, California	10/31/2007	San Jose, Private Residence	0.35	10	9	5.40	199	10.50	1.98

14	Lafayette, California	03/02/2007	Martinez, VA Medical Clinic	0.39	10	16	4.40	149	5.29	0.26

15	San Simeon, California	12/22/2003	San Luis Obispo, Rec. Center	0.40	61	7	6.40	162	13.40	9.70

16	Sierra El Mayor, Mexico	04/04/2010	Calexico Fire Station	0.40	62	10	7.20	266	40.60	13.80

17	Collins Valley, California	07/07/2010	Mountain Center, Pine Meadows R.	0.75	20	11	5.43	185	14.30	1.84

18	Big Bear, California	06/28/1992	Morongo Valley Fire Station	0.81	28	5	6.50	198	12.10	1.15

19	Nisqually, Washington	02/28/2001	Olympia, WDOT Highway Test Lab	0.82	18	59	6.80	250	16.47	4.67

20	Offshore Northern, California	01/10/2010	Ferndale, Lost Coast Ranch	0.88	36	21	6.50	352	37.20	10.96

**Table 4:** Statistics of damping global reduction factors (*R*_*ζG*_) for shears and the 0–2 range.

Earthquake	*R*_*ζG*,SAC_				*R*_*ζG*,EQ_				*R*_*ζG*,2D_				*R*_*ζG*,SDF_			
	SC1		SC2		EQ1		EQ2		2D1		2D2		SD1		SD2	
	N-S	E-W	N-S	E-W	N-S	E-W	N-S	E-W	N-S	E-W	N-S	E-W	N-S	E-W	N-S	E-W
1	0.54	0.56	0.62	0.56	0.47	0.61	0.64	0.51	0.54	0.58	0.51	0.54	0.66	0.69	0.77	0.71
2	0.9	0.93	0.88	0.76	0.87	0.93	0.88	0.88	0.92	0.95	0.88	0.85	0.99	1.01	0.98	0.97
3	0.87	0.95	0.76	0.82	0.76	0.75	0.88	0.82	0.9	0.97	0.76	0.82	0.99	0.97	0.9	0.92
4	0.74	0.59	0.67	0.8	0.76	0.59	0.79	0.84	0.70	0.60	0.73	0.75	0.90	0.70	0.94	0.94
5	0.91	0.94	0.85	0.82	0.9	0.61	0.83	0.75	0.69	0.94	0.81	0.82	0.86	0.97	0.98	0.96
6	0.48	0.79	0.75	0.57	0.57	0.42	0.64	0.86	0.4	0.73	0.76	0.67	0.76	0.7	0.79	0.76
7	0.84	0.83	0.77	0.75	0.76	0.77	0.88	0.87	0.9	0.85	0.78	0.72	0.95	0.97	0.92	0.92
8	0.74	0.61	0.86	0.64	0.68	0.48	0.81	0.84	0.74	0.66	0.66	0.82	0.74	0.58	0.97	0.79
9	0.83	0.66	0.88	0.77	0.85	0.91	0.83	0.89	0.82	0.75	0.84	0.82	0.95	0.93	0.96	0.97
10	0.79	0.96	0.88	0.89	0.9	0.78	0.87	0.88	0.8	0.96	0.9	0.9	0.86	0.97	0.99	0.97
11	0.86	0.84	0.87	0.85	0.64	0.81	0.85	0.91	0.85	0.81	0.87	0.81	0.92	0.94	0.99	0.96
12	0.83	0.57	0.82	0.6	0.85	0.59	0.74	0.68	0.86	0.75	0.78	0.81	0.95	0.97	0.92	0.76
13	0.59	0.71	0.77	0.75	0.89	0.67	0.87	0.65	0.58	0.78	0.77	0.77	0.62	0.83	0.98	0.93
14	0.63	0.77	0.89	0.89	0.84	0.91	0.73	0.9	0.6	0.77	0.88	0.93	0.66	0.84	0.97	0.97
15	0.56	0.84	0.74	0.71	0.68	0.66	0.75	0.73	0.48	0.78	0.6	0.75	0.69	0.82	0.63	0.71
16	0.49	0.59	0.53	0.57	0.84	0.55	0.49	0.66	0.5	0.6	0.57	0.58	0.52	0.53	0.91	0.48
17	0.91	0.89	0.83	0.87	0.78	0.64	0.89	0.90	0.87	0.88	0.88	0.88	0.93	0.97	0.94	0.94
18	0.86	0.62	0.67	0.62	0.59	0.63	0.51	0.56	0.64	0.50	0.64	0.66	0.58	0.5	0.9	0.99
19	0.64	0.51	0.68	0.68	0.7	0.68	0.64	0.61	0.5	0.67	0.69	0.6	0.81	0.48	0.94	0.81
20	0.77	0.91	0.62	0.85	0.54	0.75	0.69	0.67	0.73	0.67	0.56	0.54	0.51	0.87	0.71	0.6

Mean	0.74	0.75	0.77	0.74	0.74	0.69	0.76	0.77	0.70	0.76	0.74	0.75	0.79	0.81	0.91	0.85
COV	0.20	0.20	0.14	0.15	0.17	0.20	0.16	0.16	0.23	0.18	0.16	0.16	0.20	0.22	0.11	0.17

**Table 5:** Statistics of damping global reduction factors (*R*_*ζG*_) for shears and the 2–5 range.

Earthquake	*R*_*ζG*,SAC_				*R*_*ζG*,EQ_				*R*_*ζG*,2D_				*R*_*ζG*,SDF_			
	SC1		SC2		EQ1		EQ2		2D1		2D2		SD1		SD2	
	N-S	E-W	N-S	E-W	N-S	E-W	N-S	E-W	N-S	E-W	N-S	E-W	N-S	E-W	N-S	E-W
1	0.64	0.76	0.77	0.78	0.74	0.75	0.75	0.77	0.71	0.75	0.74	0.75	0.70	0.78	0.77	0.82
2	0.95	0.94	0.93	0.90	0.91	0.93	0.94	0.93	0.93	0.95	0.93	0.90	0.98	1.00	0.96	0.96
3	0.95	0.95	0.85	0.86	0.85	0.81	0.95	0.92	0.84	0.95	0.87	0.87	1.00	0.97	0.96	0.90
4	0.86	0.82	0.88	0.87	0.81	0.89	0.92	0.92	0.92	0.79	0.87	0.87	0.93	0.86	0.92	0.91
5	0.93	0.93	0.94	0.91	0.89	0.84	0.93	0.90	0.92	0.94	0.93	0.91	0.94	0.95	0.96	0.95
6	0.86	0.82	0.84	0.74	0.86	0.70	0.86	0.94	0.85	0.83	0.84	0.77	0.94	0.77	0.85	0.95
7	0.90	0.88	0.88	0.87	0.76	0.82	0.92	0.91	0.80	0.90	0.88	0.85	0.95	0.99	0.97	0.90
8	0.94	0.88	0.94	0.94	0.91	0.80	0.93	0.92	0.94	0.91	0.95	0.94	0.95	0.80	0.95	0.96
9	0.85	0.87	0.88	0.91	0.95	0.92	0.91	0.91	0.95	0.87	0.88	0.91	0.92	0.91	0.92	0.97
10	0.85	0.95	0.97	0.93	0.88	0.92	0.91	0.90	0.86	0.95	0.97	0.93	0.91	0.96	0.99	0.96
11	0.91	0.93	0.93	0.93	0.89	0.92	0.90	0.94	0.89	0.94	0.93	0.93	0.91	0.94	1.01	0.96
12	0.93	0.91	0.88	0.84	0.94	0.85	0.89	0.79	0.88	0.92	0.87	0.92	0.94	0.95	0.88	0.86
13	0.80	0.84	0.87	0.87	0.89	0.83	0.96	0.83	0.91	0.84	0.88	0.87	0.85	0.89	0.97	0.95
14	0.87	0.90	0.92	0.94	0.92	0.91	0.88	0.90	0.94	0.91	0.92	0.94	0.90	0.97	0.95	0.96
15	0.87	0.92	0.90	0.83	0.88	0.91	0.82	0.85	0.93	0.90	0.90	0.87	0.86	0.82	0.89	0.86
16	0.71	0.71	0.77	0.79	0.74	0.74	0.70	0.84	0.85	0.76	0.82	0.81	0.63	0.80	0.83	0.86
17	0.92	0.94	0.90	0.90	0.82	0.97	0.91	0.91	0.92	0.93	0.91	0.89	0.92	0.95	0.92	0.92
18	0.83	0.73	0.81	0.71	0.74	0.75	0.71	0.74	0.90	0.71	0.82	0.76	0.80	0.75	0.95	0.91
19	0.84	0.86	0.82	0.82	0.79	0.71	0.92	0.80	0.88	0.82	0.89	0.86	0.80	0.70	0.90	0.87
20	0.87	0.88	0.85	0.94	0.82	0.94	0.88	0.91	0.95	0.87	0.76	0.84	0.77	0.86	0.74	0.68

Mean	0.86	0.87	0.88	0.86	0.85	0.85	0.88	0.88	0.89	0.87	0.88	0.87	0.88	0.88	0.91	0.91
COV	0.09	0.08	0.06	0.08	0.08	0.10	0.09	0.07	0.07	0.08	0.07	0.07	0.11	0.10	0.08	0.08

**Table 6:** Statistics of global damping reduction factors (*R*_*ζG*_) for displacements and the 0–2 range.

Earthquake	*R*_*ζG*,SAC_				*R*_*ζG*,EQ_				*R*_*ζG*,2D_				*R*_*ζG*,SDF_			
	SC1		SC2		EQ1		EQ2		2D1		2D2		SD1		SD2	
	N-S	E-W	N-S	E-W	N-S	E-W	N-S	E-W	N-S	E-W	N-S	E-W	N-S	E-W	N-S	E-W
1	0.56	0.58	0.66	0.61	0.61	0.75	0.74	0.58	0.57	0.6	0.55	0.61	0.66	0.68	0.77	0.71
2	0.95	0.96	0.91	0.83	0.94	0.96	0.91	0.95	0.95	0.96	0.92	0.87	0.99	1.01	0.97	0.97
3	0.95	0.97	0.79	0.87	0.93	0.81	0.96	0.92	0.95	0.97	0.8	0.87	0.98	0.97	0.9	0.92
4	0.8	0.64	0.73	0.85	0.85	0.84	0.87	0.94	0.71	0.61	0.78	0.79	0.90	0.7	0.94	0.93
5	0.93	0.95	0.89	0.87	0.95	0.75	0.89	0.82	0.8	0.96	0.87	0.88	0.86	0.96	0.97	0.96
6	0.47	0.82	0.79	0.6	0.66	0.46	0.69	0.93	0.41	0.74	0.79	0.71	0.76	0.7	0.79	0.76
7	0.87	0.89	0.82	0.79	0.83	0.85	0.95	0.93	0.9	0.87	0.84	0.76	0.96	0.96	0.92	0.92
8	0.75	0.61	0.88	0.66	0.77	0.53	0.85	0.89	0.78	0.68	0.7	0.87	0.74	0.58	0.97	0.78
9	0.84	0.69	0.9	0.8	0.93	0.95	0.92	0.94	0.85	0.79	0.88	0.85	0.95	0.93	0.97	0.97
10	0.8	0.97	0.92	0.91	0.95	0.8	0.94	0.92	0.81	0.97	0.93	0.92	0.86	0.96	0.99	0.97
11	0.89	0.92	0.92	0.89	0.91	0.81	0.94	0.96	0.88	0.88	0.91	0.86	0.92	0.93	0.99	0.97
12	0.89	0.61	0.85	0.64	0.97	0.68	0.77	0.7	0.87	0.83	0.82	0.86	0.96	0.97	0.91	0.76
13	0.59	0.74	0.8	0.8	0.91	0.89	0.97	0.74	0.6	0.79	0.81	0.82	0.62	0.83	0.98	0.93
14	0.63	0.79	0.9	0.91	0.8	0.93	0.76	0.93	0.61	0.80	0.89	0.94	0.66	0.84	0.96	0.97
15	0.56	0.83	0.78	0.73	0.73	0.83	0.8	0.79	0.53	0.81	0.61	0.77	0.69	0.82	0.62	0.71
16	0.5	0.6	0.54	0.59	0.88	0.6	0.53	0.7	0.51	0.62	0.6	0.6	0.52	0.53	0.91	0.48
17	0.93	0.93	0.84	0.89	0.81	0.76	0.93	0.94	0.92	0.93	0.9	0.9	0.93	0.97	0.94	0.94
18	0.88	0.64	0.7	0.65	0.62	0.69	0.51	0.58	0.65	0.53	0.69	0.69	0.58	0.5	0.9	0.98
19	0.65	0.54	0.69	0.71	0.71	0.7	0.68	0.65	0.51	0.72	0.72	0.63	0.81	0.48	0.94	0.82
20	0.78	0.92	0.63	0.88	0.6	0.84	0.73	0.68	0.77	0.69	0.58	0.55	0.51	0.87	0.71	0.6

Mean	0.76	0.78	0.80	0.77	0.82	0.77	0.82	0.82	0.73	0.79	0.78	0.79	0.79	0.81	0.90	0.85
COV	0.21	0.20	0.14	0.15	0.15	0.17	0.17	0.16	0.23	0.17	0.16	0.15	0.20	0.22	0.11	0.17

**Table 7:** Statistics of global damping reduction factors (*R*_*ζG*_) for displacements and the 2–5 range.

Earthquake	*R*_*ζG*,SAC_				*R*_*ζG*,EQ_				*R*_*ζG*,2D_				*R*_*ζG*,SDF_			
	SC1		SC2		EQ1		EQ2		2D1		2D2		SD1		SD2	
	N-S	E-W	N-S	E-W	N-S	E-W	N-S	E-W	N-S	E-W	N-S	E-W	N-S	E-W	N-S	E-W
1	0.64	0.76	0.78	0.79	0.81	0.76	0.77	0.80	0.58	0.75	0.74	0.75	0.70	0.78	0.77	0.82
2	0.96	0.96	0.94	0.92	0.96	0.95	0.95	0.95	0.94	0.96	0.94	0.91	0.98	1.01	0.96	0.95
3	0.97	0.96	0.86	0.87	0.92	0.81	0.97	0.94	0.98	0.96	0.87	0.88	0.99	0.96	0.95	0.90
4	0.88	0.84	0.89	0.88	0.86	0.94	0.93	0.95	0.79	0.81	0.88	0.87	0.92	0.87	0.92	0.91
5	0.94	0.94	0.95	0.92	0.93	0.91	0.94	0.92	0.96	0.95	0.94	0.93	0.94	0.95	0.96	0.95
6	0.87	0.84	0.84	0.74	0.91	0.74	0.87	0.96	0.92	0.84	0.84	0.77	0.95	0.76	0.85	0.95
7	0.90	0.90	0.89	0.87	0.81	0.83	0.94	0.93	1.04	0.91	0.89	0.86	0.95	0.99	0.97	0.89
8	0.94	0.92	0.95	0.94	0.93	0.83	0.94	0.93	0.82	0.97	0.95	0.94	0.94	0.80	0.95	0.95
9	0.86	0.88	0.89	0.92	0.96	0.94	0.94	0.93	0.91	0.88	0.90	0.92	0.92	0.91	0.92	0.96
10	0.86	0.96	0.98	0.94	0.94	0.94	0.94	0.92	0.89	0.96	0.98	0.94	0.91	0.96	1.00	0.95
11	0.91	0.94	0.95	0.94	0.95	0.92	0.92	0.95	0.85	0.94	0.95	0.93	0.92	0.93	1.01	0.97
12	0.94	0.92	0.88	0.84	0.96	0.91	0.90	0.79	0.87	0.93	0.87	0.92	0.95	0.95	0.88	0.86
13	0.81	0.85	0.89	0.89	0.89	0.91	0.98	0.84	0.90	0.85	0.89	0.88	0.85	0.89	0.97	0.94
14	0.88	0.92	0.92	0.94	0.94	0.93	0.89	0.91	0.91	0.91	0.93	0.94	0.90	0.97	0.95	0.96
15	0.90	0.93	0.91	0.83	0.90	0.91	0.83	0.85	0.95	0.93	0.90	0.87	0.87	0.82	0.89	0.86
16	0.71	0.71	0.77	0.79	0.80	0.76	0.69	0.85	0.75	0.76	0.83	0.82	0.63	0.80	0.83	0.86
17	0.92	0.97	0.90	0.91	0.87	0.99	0.92	0.92	0.92	0.96	0.91	0.90	0.91	0.95	0.92	0.92
18	0.84	0.73	0.81	0.71	0.76	0.75	0.70	0.74	0.68	0.71	0.82	0.76	0.80	0.75	0.95	0.91
19	0.85	0.86	0.83	0.82	0.79	0.70	0.93	0.80	0.92	0.83	0.89	0.87	0.80	0.70	0.90	0.87
20	0.87	0.88	0.85	0.94	0.86	0.94	0.89	0.91	0.84	0.86	0.76	0.83	0.77	0.86	0.73	0.68

Mean	0.87	0.88	0.88	0.87	0.89	0.87	0.89	0.89	0.87	0.88	0.88	0.87	0.88	0.88	0.91	0.90
COV	0.09	0.09	0.07	0.08	0.07	0.10	0.09	0.07	0.12	0.09	0.07	0.07	0.11	0.10	0.08	0.08

**Table 8:** Statistics of local damping reduction factors (*R*_*ζL*_) for the 0–2 range.

			*R*_*ζL*,SAC_				*R*_*ζL*,EQ_				*R*_*ζL*,2D_				*R*_*ζL*,SDF_			
Parameter			SC1		SC2		EQ1		EQ2		2D1		2D2		SD1		SD2	
			N-S	E-W	N-S	E-W	N-S	E-W	N-S	E-W	N-S	E-W	N-S	E-W	N-S	E-W	N-S	E-W
Axial	Ext.	Mean	0.63	0.63	0.62	0.53	0.45	0.48	0.55	0.58	0.44	0.53	0.57	0.69	0.44	0.48	0.59	0.62
		COV	0.27	0.26	0.24	0.29	0.39	0.38	0.32	0.33	0.30	0.25	0.34	0.22	0.44	0.35	0.34	0.32
	Int.	Mean	0.42	0.42	0.50	0.49	0.43	0.47	0.52	0.47	0.35	0.37	0.40	0.46	0.42	0.46	0.49	0.54
		COV	0.43	0.42	0.35	0.35	0.40	0.40	0.32	0.36	0.33	0.41	0.41	0.38	0.42	0.44	0.33	0.38

Moment	Ext.	Mean	0.77	0.72	0.81	0.80	0.77	0.72	0.81	0.80	0.44	0.75	0.44	0.80	0.79	0.79	0.90	0.90
		COV	0.23	0.23	0.14	0.14	0.23	0.23	0.14	0.14	0.30	0.21	0.30	0.17	0.20	0.20	0.11	0.11
	Int.	Mean	0.77	0.71	0.81	0.80	0.77	0.71	0.81	0.80	0.35	0.76	0.35	0.80	0.79	0.79	0.91	0.91
		COV	0.23	0.22	0.14	0.15	0.23	0.22	0.14	0.15	0.33	0.22	0.33	0.18	0.20	0.20	0.11	0.11

**Table 9:** Statistics of local damping reduction factors (*R*_*ζL*_) for the 2–5 range.

			*R*_*ζL*,SAC_				*R*_*ζL*,EQ_				*R*_*ζL*,2D_				*R*_*ζL*,SDF_			
Parameter			SC1		SC2		EQ1		EQ2		2D1		2D2		SD1		SD2	
			N-S	E-W	N-S	E-W	N-S	E-W	N-S	E-W	N-S	E-W	N-S	E-W	N-S	E-W	N-S	E-W
Axial	Ext.	Mean	0.84	0.87	0.79	0.73	0.75	0.77	0.79	0.81	0.84	0.85	0.88	0.86	0.72	0.77	0.81	0.82
		COV	0.13	0.14	0.15	0.13	0.15	0.13	0.20	0.18	0.11	0.11	0.09	0.10	0.09	0.13	0.13	0.13
	Int.	Mean	0.77	0.75	0.81	0.81	0.75	0.77	0.79	0.79	0.77	0.76	0.74	0.74	0.73	0.75	0.75	0.76
		COV	0.18	0.19	0.15	0.16	0.15	0.13	0.19	0.19	0.13	0.12	0.15	0.16	0.19	0.11	0.15	0.16

Moment	Ext.	Mean	0.87	0.86	0.89	0.89	0.87	0.87	0.89	0.89	0.87	0.87	0.91	0.89	0.88	0.88	0.91	0.92
		COV	0.09	0.10	0.09	0.09	0.08	0.08	0.10	0.09	0.08	0.09	0.08	0.08	0.10	0.11	0.08	0.08
	Int.	Mean	0.87	0.86	0.89	0.89	0.87	0.87	0.89	0.89	0.87	0.87	0.90	0.89	0.88	0.88	0.92	0.92
		COV	0.10	0.09	0.09	0.09	0.08	0.08	0.09	0.09	0.08	0.09	0.08	0.08	0.11	0.11	0.08	0.08

**Table 10:** Statistics of global yielding reduction factors (*R*_PG_) for shears and *ζ* = 2%.

Earthquake	*R*_PG,SAC_				*R*_PG,EQ_				*R*_PG,2D_				*R*_PG,SDF_			
	SC1		SC2		EQ1		EQ2		2D1		2D2		SD1		SD2	
	N-S	E-W	N-S	E-W	N-S	E-W	N-S	E-W	N-S	E-W	N-S	E-W	N-S	E-W	N-S	E-W
1	0.97	0.97	0.89	0.93	0.99	0.97	0.98	0.91	0.72	0.66	0.50	0.72	1.00	1.00	1.01	1.03
2	0.97	0.87	0.75	0.85	0.87	0.81	0.72	0.82	0.71	0.72	0.67	0.79	0.95	1.00	1.00	1.01
3	0.94	1.03	0.82	0.75	0.94	0.95	0.86	0.95	0.91	0.79	0.66	0.72	1.00	0.97	1.01	1.01
4	0.98	0.84	0.94	0.87	0.99	1.03	0.97	0.90	0.61	0.63	0.72	0.79	1.00	1.00	1.01	0.97
5	0.93	0.74	0.79	0.83	0.79	0.74	0.78	0.98	0.77	0.73	0.78	0.83	1.00	1.00	1.01	1.01
6	0.94	0.96	0.90	0.81	0.94	0.98	0.84	0.85	0.77	0.77	0.66	0.69	1.00	1.00	1.01	1.01
7	0.96	1.07	0.92	0.90	0.98	0.92	0.76	0.80	0.82	0.86	0.67	0.71	1.00	1.00	1.00	1.00
8	0.91	1.11	0.76	0.80	0.92	0.83	0.75	0.78	0.73	0.78	0.75	0.76	1.00	1.00	1.00	1.01
9	0.92	0.99	0.86	0.83	1.00	0.88	0.95	0.82	0.74	0.62	0.80	0.77	1.00	1.00	1.00	1.01
10	0.83	0.71	1.15	0.85	0.97	0.76	0.72	0.76	0.80	0.70	0.79	0.79	1.00	1.00	0.99	1.00
11	0.77	0.75	0.90	0.83	0.96	0.95	0.75	0.65	0.72	0.80	0.76	0.83	1.00	1.00	1.00	1.01
12	1.02	0.81	0.98	0.92	1.01	0.84	0.74	0.90	0.72	0.76	0.70	0.79	1.00	1.00	1.02	1.01
13	1.00	0.83	0.88	0.82	0.88	0.91	1.06	0.94	0.75	0.80	0.66	0.81	0.94	1.00	1.00	1.00
14	0.81	0.70	0.73	0.78	0.84	0.79	0.73	0.74	0.74	0.71	0.70	0.76	1.00	1.00	1.01	1.02
15	0.96	0.96	0.78	0.90	0.74	1.03	0.79	0.91	0.73	0.85	0.69	0.79	1.00	1.00	1.00	1.00
16	0.97	0.99	0.98	0.84	1.00	0.91	1.00	0.98	0.64	0.59	0.72	0.74	1.00	1.00	1.01	1.00
17	0.74	0.87	0.75	0.84	0.83	1.06	0.93	0.81	0.78	0.76	0.72	0.82	1.00	0.96	1.01	1.01
18	0.95	0.98	0.98	0.96	0.99	1.00	0.98	0.98	0.69	0.56	0.71	0.62	0.97	1.00	1.03	0.98
19	0.96	0.88	0.75	0.95	0.83	0.96	1.26	0.97	0.65	0.67	0.66	0.75	1.00	1.00	1.01	1.00
20	1.05	0.75	0.96	1.08	0.84	1.00	0.87	1.01	0.70	0.65	0.62	0.70	1.00	1.00	1.01	1.01

Mean	0.93	0.89	0.87	0.87	0.92	0.92	0.88	0.87	0.74	0.72	0.70	0.76	1.00	1.00	1.01	1.01
COV	0.09	0.14	0.12	0.09	0.09	0.11	0.18	0.11	0.09	0.12	0.10	0.07	0.02	0.01	0.01	0.01

**Table 11:** Statistics of global yielding reduction factors (*R*_PG_) for shears and *ζ* = 5%.

Earthquake	*R*_PG,SAC_				*R*_PG,EQ_				*R*_PG,2D_				*R*_PG,SDF_			
	SC1		SC2		EQ1		EQ2		2D1		2D2		SD1		SD2	
	N-S	E-W	N-S	E-W	N-S	E-W	N-S	E-W	N-S	E-W	N-S	E-W	N-S	E-W	N-S	E-W
1	0.99	1.00	0.93	0.95	0.98	0.98	0.98	0.94	0.65	0.51	0.57	0.78	1.00	1.00	1.03	1.00
2	0.97	1.33	0.74	0.80	1.06	1.09	0.67	1.02	0.62	0.72	0.63	0.78	1.00	1.00	1.01	1.02
3	0.95	0.96	0.82	0.82	0.69	0.98	0.80	0.93	0.96	0.80	0.67	0.71	1.00	1.00	1.01	1.00
4	1.00	0.81	0.96	0.87	1.00	1.01	1.03	0.87	0.56	0.63	0.71	0.79	1.00	1.00	0.97	1.00
5	1.00	0.66	0.84	0.79	1.02	1.00	1.05	0.96	0.75	0.60	0.72	0.80	0.97	1.00	1.01	1.00
6	0.89	0.95	0.93	0.85	1.02	0.93	0.77	0.77	0.77	0.63	0.69	0.78	1.00	0.97	1.01	1.00
7	0.99	1.31	0.80	0.88	0.81	0.85	0.92	0.81	0.67	0.77	0.65	0.71	1.00	1.00	1.00	1.00
8	0.88	1.12	0.77	0.71	1.00	0.99	0.76	0.74	0.68	0.76	0.71	0.73	1.00	1.00	1.01	1.00
9	0.87	1.06	0.86	0.79	0.95	0.93	0.88	0.81	0.67	0.61	0.77	0.74	1.00	1.00	1.01	1.00
10	0.91	0.84	1.05	0.82	0.94	0.69	0.74	0.75	0.69	0.67	0.67	0.75	1.00	1.00	1.00	0.98
11	0.78	0.72	0.89	0.80	0.94	0.92	0.80	1.21	0.66	0.67	0.71	0.79	0.95	1.00	1.01	1.00
12	0.95	0.69	0.99	0.94	1.02	0.99	0.76	0.97	0.65	0.64	0.68	0.78	1.00	0.99	1.01	1.01
13	1.00	0.90	0.88	0.81	0.96	0.98	1.10	1.01	0.72	0.59	0.63	0.81	0.98	1.00	1.00	1.00
14	0.71	0.68	0.73	0.69	0.89	0.89	0.70	0.78	0.67	0.68	0.66	0.74	1.00	1.00	1.02	1.00
15	0.92	1.02	0.76	0.91	0.76	1.00	0.86	0.93	0.68	0.69	0.66	0.79	1.00	1.00	1.00	1.01
16	0.98	1.00	0.97	0.87	1.00	0.94	1.01	0.98	0.66	0.62	0.76	0.76	1.00	1.00	1.00	1.00
17	0.71	0.95	0.74	0.83	0.80	1.12	0.91	0.80	0.69	0.71	0.69	0.80	0.96	1.00	1.01	1.00
18	0.93	1.04	0.99	1.00	1.00	0.99	0.97	0.97	0.65	0.71	0.74	0.71	1.00	0.96	0.98	1.03
19	0.95	0.92	0.79	0.99	0.74	0.96	1.11	0.92	0.62	0.59	0.66	0.75	1.00	1.00	1.00	1.00
20	0.89	0.69	0.96	0.90	0.91	1.00	0.86	0.90	0.68	0.51	0.71	0.73	1.00	1.00	1.01	1.00

Mean	0.91	0.93	0.87	0.85	0.92	0.96	0.88	0.90	0.69	0.66	0.68	0.76	1.00	1.00	1.01	1.00
COV	0.10	0.21	0.11	0.10	0.12	0.09	0.15	0.13	0.12	0.12	0.07	0.04	0.03	0.02	0.01	0.10

**Table 12:** Statistics of local yielding local reduction factors (*R*_PL_) for *ζ* = 2%.

			*R*_PL,SAC_				*R*_PL,EQ_				*R*_PL,2D_				*R*_PL,SDF_			
Parameter			SC1		SC2		EQ1		EQ2		2D1		2D2		SD1		SD2	
			N-S	E-W	N-S	E-W	N-S	E-W	N-S	E-W	N-S	E-W	N-S	E-W	N-S	E-W	N-S	E-W
Axial	Ext.	Mean	0.60	0.55	0.96	0.87	0.32	0.30	0.22	0.23	0.65	0.57	0.53	0.56	0.83	0.71	0.40	0.38
		COV	0.17	0.18	0.31	0.29	0.48	0.63	0.37	0.36	0.20	0.26	0.14	0.13	0.23	0.23	0.36	0.25
	Int.	Mean	0.32	0.25	0.24	0.20	0.31	0.30	0.21	0.26	0.97	1.08	0.73	0.96	1.01	0.80	0.63	0.46
		COV	0.44	0.36	0.30	0.30	0.48	0.63	0.45	0.40	0.07	0.22	0.34	0.20	0.26	0.25	0.32	0.28

Moment	Ext.	Mean	0.97	1.10	0.91	1.06	0.86	0.85	0.87	1.00	0.69	0.66	0.67	0.79	1.00	1.00	1.01	1.01
		COV	0.13	0.13	0.07	0.15	0.17	0.18	0.21	0.12	0.10	0.11	0.11	0.07	0.01	0.00	0.01	0.01
	Int.	Mean	0.97	1.10	0.90	1.06	0.85	0.87	0.89	0.98	0.74	0.74	0.77	0.87	0.98	0.99	1.01	1.01
		COV	0.13	0.13	0.07	0.15	0.17	0.19	0.21	0.14	0.18	0.20	0.13	0.06	0.01	0.01	0.01	0.01

**Table 13:** Statistics of local yielding local reduction factors (*R*_PL_) for *ζ* = 5%.

			*R*_PL,SAC_				*R*_PL,EQ_				*R*_PL,2D_				*R*_PL,SDF_			
Parameter			SC1		SC2		EQ1		EQ2		2D1		2D2		SD1		SD2	
			N-S	E-W	N-S	E-W	N-S	E-W	N-S	E-W	N-S	E-W	N-S	E-W	N-S	E-W	N-S	E-W
Axial	Ext.	Mean	0.53	0.49	1.09	0.96	0.25	0.25	0.20	0.24	0.58	0.49	0.52	0.56	0.76	0.61	0.36	0.33
		COV	0.25	0.23	0.29	0.30	0.55	0.70	0.35	0.51	0.28	0.16	0.16	0.07	0.21	0.22	0.37	0.23
	Int.	Mean	0.23	0.22	0.22	0.19	0.25	0.25	0.17	0.25	0.99	1.00	0.67	0.94	1.10	0.70	0.59	0.45
		COV	0.52	0.34	0.36	0.25	0.55	0.70	0.28	0.40	0.14	0.22	0.32	0.19	0.27	0.23	0.25	0.36

Moment	Ext.	Mean	1.00	1.19	0.92	1.06	0.89	0.88	0.91	0.99	0.60	0.56	0.66	0.80	1.00	1.00	1.01	1.01
		COV	0.19	0.17	0.08	0.10	0.14	0.14	0.19	0.15	0.16	0.17	0.09	0.03	0.01	0.01	0.01	0.01
	Int.	Mean	0.96	1.19	0.92	1.06	0.89	0.88	0.90	1.00	0.72	0.66	0.77	0.88	1.00	1.00	1.01	1.01
		COV	0.19	0.17	0.08	0.10	0.14	0.14	0.16	0.15	0.28	0.22	0.08	0.03	0.01	0.01	0.01	0.01
